# Mina Svar: A tool for measuring social impact

**DOI:** 10.1093/eurpub/ckac131.248

**Published:** 2022-10-25

**Authors:** R Salari, J Westberg, Å Moum, Youth in Save the Children programs, M Thell, O Dyar, A Sarkadi

**Affiliations:** 1 Child Health and Parenting, Uppsala University, Uppsala, Sweden; 2 Save the Children, Stockholm, Sweden

## Abstract

**Background:**

Each year, numerous initiatives are carried out to improve the outcomes for youth living in vulnerable areas. However, the impact of these initiatives is rarely measured, partly because there is no reliable, valid, relevant and easy-to-use tool available to measure the impact of social investments from the youth perspective. Mina Svar is an app co-created with youth that aims to address this gap.

**Methods and results:**

Save the Children led the collaboration in defining a measurement framework. Experts from Save the Children, researchers from Linköping University, and representatives from Accenture, Skandia and Apoteket as well as youth themselves were involved in an iterative process. Five interconnected areas were identified as central: democracy and influence, education, work, housing and neighbourhood, and health. The first version of Mina Svar included 34 items. Researchers from Uppsala University were involved to help with testing the psychometric properties of Mina Svar in a sample of 237 youths. We examined the tool’s internal consistency, content validity and structure validity. Analyses showed that all the subscales except democracy and influence had good internal consistency (0.70 and higher). However, inspection of individual items revealed that several items lacked clarity and many items did not comprehensively reflect the related constructs as intended. Confirmatory factor analysis suggested a poor fit for the proposed model (CFI = 0.52 TLI = 0.56, and RMSEA = 0.103). Currently, we are going through a second iterative process to increase the reliability and validity of Mina Svar. The work involves refining the framework, rewriting ambiguous items, generating new items, and re-examining the psychometric properties of the revised version.

**Conclusions:**

Mina Svar is a promising short multidimensional survey tool which offers a potential solution to tackle the problem with measuring the impact of social investments from the youth perspective.

**Key messages:**

• Mina Svar is an app-based assessment tool co-created with youths living in vulnerable areas.

• Mina Svar is a promising short multidimensional tool which offers a potential solution to tackle the problem with measuring the impact of social investments.

